# Involvement of fear, incompleteness, and disgust during symptoms of pediatric obsessive–compulsive disorder

**DOI:** 10.1007/s00787-020-01514-7

**Published:** 2020-03-24

**Authors:** Matti Cervin, Sean Perrin, Elin Olsson, Emma Claesdotter-Knutsson, Magnus Lindvall

**Affiliations:** 1 Department of Clinical Sciences Lund, Child and Adolescent Psychiatry, Faculty of Medicine, Lund University, Sofiavägen 2D, 22241 Lund, Sweden; 2 Skåne Child and Adolescent Psychiatry, Lund, Sweden; 3 Department of Psychology, Lund University, Lund, Sweden

**Keywords:** OCD, Children, Emotion, Motivation, Fear, Incompleteness, Disgust

## Abstract

Fear has been assigned a central role in models of obsessive–compulsive disorder (OCD), but empirical investigations into the emotions that underpin OCD symptoms are few, especially in pediatric samples. Using validated, clinician-led structured interviews, 124 youth with OCD reported on the presence and severity of symptoms across the main symptom dimensions of OCD (aggressive, symmetry, contamination) and the degree to which fear, incompleteness, and disgust accompanied these symptoms. For comparison purposes, the degree of fear, incompleteness, and disgust during symptoms was obtained also from youth with social anxiety disorder (SAD; *n* = 27) and generalized anxiety disorder (GAD*; n* = 28). Participants with OCD reported that all three emotions were involved in their symptoms; however, fear was most strongly linked to aggressive symptoms, incompleteness to symmetry symptoms, and disgust to contamination symptoms. Incompleteness differentiated youth with OCD from those with SAD and GAD. No differences for these emotions were found for youth with OCD with versus without the tic-disorder subtype or comorbid autism. A positive association between incompleteness and self-reported hoarding emerged among youth with OCD. Further studies of the emotional architecture of pediatric OCD, and its relationship to etiology and treatment, are warranted.

## Introduction

Pediatric obsessive–compulsive disorder (OCD) is a heterogeneous and disabling condition affecting 2–4% of children and adolescents [1, 2]. Cognitive behavioral therapy (CBT) and selective serotonin reuptake inhibitors (SSRIs) are first-line treatments but yield sub-optimal effects for a significant proportion of patients [3] and the prognosis for patients who retain clinically significant symptoms of OCD is often poor [4–7]. There is little consistent information about who is most likely to benefit from these treatments and further improvements in outcomes will require a better understanding of the etiological and maintaining factors that underpin this disorder [8–10].

While numerous psychological models have been posited to explain the onset and maintenance of OCD, among the most well-researched and influential are cognitive behavioral models of OCD (for a discussion, see [11]). Briefly, cognitive behavioral models view obsessions as transient, intrusions of fearful images or thoughts of the kind that are common in the general population (e.g., the thought that something bad might happen to a loved one while walking to school). If the individual focuses on these intrusions and appraises them such that the intrusion now means the feared outcome is more likely, and the individual is responsible for preventing the feared outcome, there is an increase in fear, distress, and an urge to neutralize this thought and prevent the feared outcome. Over time, such appraisals and the resulting fear response drive the development of a wide range of neutralizing (escape) and avoidance behaviors [11]. From this perspective, OCD and anxiety disorders are similar in that they are driven by exaggerated fears about the probability of harm and maintained by the excessive use of idiosyncratic safety-seeking behaviors.

Although many OCD patients report fear and anxiety as part of their obsessions and compulsions, a large proportion do not. This is one reason why OCD was removed from the anxiety disorders chapter in DSM-5 and placed alongside other conditions characterized not by a single dominant emotional response, but by the presence of compulsivity and repetitive behaviors [12]. Indeed, since Janet described OCD in the beginning of the twentieth century [13], clinicians and researchers have recognized that emotions (or emotion-like states) other than fear are experienced during obsessions and compulsions, and as such may be important to the etiology and treatment of OCD. Examples of such emotion-related experiences include pathological doubt, anger, disgust, and incompleteness or not-just-right experiences (NJRE) [14–19]. This study concerns itself with the role of fear, incompleteness, and disgust in the experience of OCD symptoms in children and adolescents.

Recognizing that the ritualized and repetitive nature of compulsions, and an often irrational need for (or preoccupation with) symmetry, appears to distinguish OCD from the anxiety disorders, Summerfeldt et al. [15, 20] proposed the Core Dimensions Model of OCD. Central to this model is the notion that both fear (termed harm avoidance in the model) and a felt sense of incompleteness are the core motivational processes underpinning OCD symptoms [16]. The emotional experience of incompleteness refers to an inner sensation of things being ‘not just right’ and an emotional urge to reach a sense of completeness [21]. There is a small but growing body of evidence which suggests that a felt sense of incompleteness, both as an overall trait and during OCD symptoms, is elevated among adults [16] and youth with OCD [22], and that incompleteness, compared to fear, may be uniquely associated with OCD in both children and adults [18, 22, 23]. Furthermore, a recent study found that higher levels of incompleteness during OCD symptoms was associated with poorer OCD outcomes in youth treated for this disorder [24].

Disgust has long been recognized as a potential contributor to the severity and maintenance of contamination-related fears and avoidance behaviors that occur as part of OCD and social and specific phobias [25]. There is a growing body of evidence suggesting that a general proneness or sensitivity for disgust is associated with more severe OCD symptoms, especially those involving the theme of contamination [14, 26, 27]. This association is also found in clinical and non-clinical samples of youth, with changes in disgust sensitivity during OCD treatment being positively correlated with reductions in OCD symptoms [14, 22, 28, 29]. However, as disgust proneness/sensitivity is also correlated with symptoms of anxiety, and as anxiety symptoms also tend to decrease during OCD treatment, the extent to which disgust plays a primary or secondary (to anxiety) role in OCD remains unclear [30].

Taken together, there is increasing evidence to suggest that, in addition to fear, incompleteness and to some extent disgust are elevated among individuals with OCD, and often accompany OCD symptoms, suggesting that multiple emotion-related motivators may be present in OCD. The strength of a multi-emotion based perspective on OCD is that topographically identical symptoms (e.g., excessive handwashing or repetitive checking) may be motivated and/or driven by different emotions. For example, hand washing may be motivated by a desire to reduce the risk of becoming ill, by a strong sense of disgust, or by an urge to reach a sense of completeness in the handwashing ritual. Importantly, it has been argued that these emotion-related motivators reflect not only conceptual or phenomenological differences, but also differences in more basic psychological and neural processes, which could shed new light on the etiology and treatment of OCD [31–33]. Hence, further studies are needed of the emotions that accompany OCD symptoms, and how these relate to the severity and persistence of the disorder, particularly in children and adolescents. In addition, the relationship between fear, incompleteness, disgust, and OCD symptoms has most often been examined using self-report measures of these emotions at an overall, trait-like level (i.e., a general proneness for fear, incompleteness, and disgust). Although useful, this is not the same as asking patients about the extent to which feelings of fear, disgust, or incompleteness are experienced during or directly involved in their OCD symptoms.

It is important to note that OCD symptoms tend to cluster within several, temporally stable symptom dimensions, with the three most commonly observed being the aggressive, symmetry, and contamination-related dimensions [34, 35]. Studies suggest that these dimensions involve different neural substrates [36, 37] and possibly different etiologies [38]. As such, these dimensions provide a useful framework in which emotion-involvement in OCD symptoms can be studied [39]. Finally, given the overlap at the conceptual, etiological, and phenomenological level between OCD and the anxiety disorders [40, 41], studies examining potential emotion-related motivators in individuals with OCD should include anxiety disordered comparison groups to help distinguish between potentially OCD-specific, anxiety-specific, and transdiagnostic emotional processes.

The present study was undertaken to address the above gaps in the current literature. Our primary aim was to examine the extent to which fear, incompleteness, and disgust accompanied current OCD symptoms in a large and diverse sample of treatment-seeking youth with OCD, as a disorder overall, and in relation to its three major symptom dimensions (aggressive, symmetry, contamination). In a preliminary fashion, and for comparison purposes, we undertook a similar examination with youth seeking treatment for social anxiety disorder (SAD) and generalized anxiety disorder (GAD), and without a diagnosis of OCD. For participants with OCD, we also investigated whether fear, incompleteness, and disgust were more or less associated with certain forms of comorbidity that have been shown to be associated with the severity of OCD, namely hoarding, tics, and autism spectrum disorder [42–44].

## Method

### Participants

Participants were 179 youth (aged 7–17 years) consecutively referred for either OCD or anxiety to a specialized child and adolescent mental health clinic in Lund (Southern Sweden) between January 2016 and December 2018. The study was conducted as part of a larger, externally funded project about cognitive and emotional mechanisms in pediatric OCD. All patients that met eligibility criteria and consented to participate while the study ran were included. Of the 179 included participants, 124 youth had a diagnosis of OCD, either primary (*n* = 115; 93%) or secondary (*n* = 9; 7%); 27 had a current primary diagnosis of SAD; and 28 had a current primary diagnosis of GAD. Only participants with SAD/GAD who did not meet diagnostic criteria for a current diagnosis of OCD were included in the study. Of the participants with OCD as a secondary diagnosis, three had primary depression, three primary GAD, one primary separation anxiety disorder, and two primary bipolar disorder. Sociodemographic and clinical characteristics of the diagnostic groups, with the OCD participants divided along their primary symptom dimensions are presented in Table 1.

**Table 1:** Sociodemographic and clinical characteristics across groups

	Aggressive OCD *n* = 50	Symmetry OCD *n* = 31	Contamination OCD *n* = 39	SAD *n* = 27	GAD *n* = 28
Sociodemographic characteristics					
Age, years, *M* (*SD*)	13.51 (2.91)	12.66 (2.66)	13.70 (2.62)	15.90 (1.33)	14.83 (2.35)
Female, *n* (*%*)	32 (64.0%)	22 (71.0%)	21 (53.8%)	24 (88.9%)	25 (89.3%)
Living with both parents, *n* (*%*)	35 (70.0%)	20 (64.5%)	23 (59.0%)	12 (44.4%)	18 (64.3%)
Mothers, university education, *n* (*%*)	38 (79.2%)	23 (74.2%)	27 (69.2%)	14 (58.3%)	12 (46.2%)
Fathers, university education, *n* (*%*)	26 (54.2%)	16 (51.6%)	23 (59.0%)	14 (58.3%)	11 (42.3%)
Family economy, good or very good, *n* (*%*)	37 (78.7%)	24 (77.4%)	28 (77.8%)	16 (84.2%)	15 (60.0%)
Clinical characteristics					
OCD, *n* (*%*)	50 (100%)	31 (100%)	39 (100%)	0 (0%)	0 (0%)
GAD, *n* (*%*)	15 (30%)	10 (32.3%)	7 (17.9%)	6 (22.2%)	28 (100%)
SAD, *n* (*%*)	7 (14.9%)	2 (6.5%)	2 (5.1%)	27 (100%)	7 (28.0%)
Any anxiety disorder, *n* (*%*)	28 (56.0%)	17 (54.8%)	14 (35.9%)	27 (100%)	28 (100%)
Major depression, *n* (*%*)	4 (8.0%)	3 (9.7%)	3 (7.7%)	12 (44.4%)	8 (28.6%)
ADHD, *n* (*%*)	6 (12.0%)	5 (16.1%)	9 (23.1%)	0 (0%)	4 (14.3%)
Autism, *n* (*%*)	3 (6.0%)	0 (0%)	5 (12.8%)	0 (0%)	1 (3.6%)
CY-BOCS, *M* (*SD*)	24.22 (4.77)	23.51 (3.60)	21.63 (4.05)	–	–
AQ10, *M* (*SD*)	2.54 (2.40)	2.42 (1.92)	2.44 (1.96)	–	–
OCI-CV Hoarding, *M* (*SD*)	2.10 (1.73)	2.19 (1.52)	0.77 (1.17)	–	–
Age of OCD symptom onset, *M* (*SD*)	8.70 (2.92)	7.42 (2.14)	8.79 (3.16)	–	–
First-degree relative with OCD, *n* (*%*)	10 (20.4%)	6 (20.0%)	11 (28.9%)	–	–
Lifetime history of tic disorder, *n* (*%*)	10 (20.4%)	11 (35.5%)	12 (30.8%)	–	–

Missing data for parental education and family economy was 2.7% for the OCD group, 17.3% for the SAD group, and 8.3% for the GAD group

*OCD* obsessive–compulsive disorder, *GAD* Generalized anxiety disorder, *SAD* social anxiety disorder, *CY-BOCS* Children’s Yale–Brown Obsessive Compulsive Scale, *CGI-S* Clinical Global Impression-Severity, *CGI-I* Clinical Global Impression-Improvement

## Measures

### Diagnostic status

Diagnostic status was assessed with the Mini International Neuropsychiatric Interview for Children and Adolescents which is a reliable and valid tool to establish mental disorders in youth [45]. The diagnoses of OCD, SAD, and GAD were established according to DSM-5 criteria.

### OCD severity at disorder and symptom dimension level

Overall OCD symptom severity was assessed with the clinician-administered, 10-item Children’s Yale-Brown Obsessive Compulsive Scale (CY-BOCS) [46], a measure that assesses overall time, interference, control, resistance, and distress related to obsessions and compulsions and yields an overall score of 0 to 40 with higher scores indicating more severe OCD. The measure has repeatedly been found to be a reliable and valid measure of the severity of pediatric OCD [47].

To assess symptom severity for each of the major symptom dimensions of OCD, we used an updated version of the clinician-administered, Dimensional Yale–Brown Obsessive Compulsive Scale (DY-BOCS [48]). The DY-BOCS is an interview that screens for and rates the severity of symptoms for aggressive, sexual/religious, symmetry, contamination, and miscellaneous obsessions and related compulsions. Each symptom dimension is rated as absent/present, and if present, symptom severity is rated according to three 0–5 items assessing time, distress, and interference. Obsessions, compulsions, and avoidance are rated together. For each symptom dimension, a total score is computed (0–15), with higher scores indicating more severe symptoms. The DY-BOCS has been found to be a valid and reliable measure of OCD symptom dimensions in youth and adults with OCD [48, 49].

### Fear, incompleteness, and disgust as motivators of OCD, SAD, and GAD

To assess the degree to which fear, incompleteness, and disgust accompanied symptoms, all participants were interviewed with the clinician-administered Obsessive–Compulsive Core Dimensions Interview (OC-CDI [15]). Originally, the OC-CDI assessed only the roles of fear and incompleteness, and not disgust, and in relation to OCD symptoms only, not anxiety. The interview has been expanded to assess the role of disgust during OCD symptoms, and as such it has been shown to possess good psychometric properties and adequate construct validity in a large sample of youth with OCD [50].

In the present study, youth with OCD were asked to rate the degree to which they experienced fear, incompleteness, and disgust during their current symptoms of OCD, grouped within symptom dimensions, as defined by their responses to the DY-BOCS. SAD and GAD participants rated these same emotions in relation to their current SAD and GAD symptoms, as identified during the MINI-KID interview. In all OC-CDI assessments, the interviewer asked the participant to describe the most recent symptom episode of which they had a clear memory (e.g., going to a party, worrying about the future, touching dirty dishes and hand washing). Next, they were given detailed explanations of the emotional experiences of fear, incompleteness, and disgust, and were asked to rate the extent to which each of these were involved during their symptoms. To facilitate their ratings, a graphical version of the rating scale was placed in front of them, showing the ratings going from 0 (not all) to 4 (extremely).

### Primary symptom dimension and global emotion score

Because we were interested in potential differences between emotional involvement across the major symptom dimensions of OCD, each OCD participant was assigned a primary symptom dimension based on their highest DY-BOCS symptom dimension score. If a participant had identical scores in two or more dimensions, the dimension with the most debilitating impact was established as the primary dimension. Few participants had sexual/religious (*n* = 2) and miscellaneous symptoms (*n* = 8) as their primary dimension, and these dimensions were excluded from dimension-specific analyses. The primary symptom dimensions in youth with OCD were: primary aggressive (*n* = 50), primary symmetry (*n* = 31), and primary contamination (*n* = 39).

For OCD participants with symptoms in several different DY-BOCS dimensions, a global score for fear, incompleteness, and disgust were computed. We identified the symptom dimensions with a severity score of ≥ 5 (i.e., clinically relevant symptoms) and used the highest fear, incompleteness, and disgust score across these clinically significant dimensions as their global fear, incompleteness, and disgust score for the OC-CDI. For example, a patient with clinically relevant taboo symptoms (i.e., a DY-BOCS severity score for this dimension of ≥ 5), motivated by fear (an OC-CDI rating of 3), but not by incompleteness or disgust (OC-CDI ratings of 0), and who also had clinically relevant symmetry/ordering symptoms motivated by disgust (an OC-CDI rating of 4), but not by fear or incompleteness (OC-CDI ratings of 0), were assigned a global score fear rating of 3, a global disgust rating of 4, and a global incompleteness rating of 0.

### Hoarding, tic disorder, and autism

Hoarding symptoms were assessed via the hoarding subscale of the Obsessive Compulsive Inventory—Child Version (OCI-CV), a three-item self-report scale on severity of hoarding symptoms with sound psychometric properties [51]. Lifetime history of tic disorder was assessed using the MINI-KID. A lifetime history of tic disorder was established during the MINI-KID interview. Regarding autism, participants either had a diagnosis when recruited or received one as part of their care and according to international guidelines for diagnosing autism in youth. Autistic traits were assessed using the 10-item, parent-report Autism Spectrum Quotient (AQ10) [52].

### Procedure and ethics

All assessments were carried out by clinical psychologists working within the anxiety and OCD team at the clinic. The study was reviewed and approved by the regional ethical review board at Lund University (Dnr 2015/663) and was performed in accordance with the 1964 Declaration of Helsinki and its later amendments. All participants and their caregivers provided written informed consent.

### Statistical analysis

Differences for fear, incompleteness, and disgust across diagnostic/symptom groups were investigated via three analyses of covariance models (ANCOVAs), with group (i.e., aggressive OCD, symmetry OCD, contamination OCD, SAD, and GAD) as the independent variable and the OC-CDI scores for fear, incompleteness, and disgust, respectively, as the dependent variable. Age and gender were included as covariates in all models. Follow-up tests on the age- and gender-adjusted marginal means were employed to explore specific group differences. Because of non-normal distributions and different sample sizes, we also carried out non-parametric Kruskall-Wallis *H* tests to examine possible group differences.

The relationship between emotion and symptom dimension severity was evaluated via three separate multiple linear regressions, with OCD symptom dimension severity as the dependent variable, and fear, incompleteness, disgust ratings for that specific dimension as independent variables. Age and gender were included as covariates in all models. In these analyses, we included all youth with OCD who affirmed clinically significant symptoms within a symptom dimension (aggressive symptoms: *n* = 84; symmetry symptoms: *n* = 80; contamination symptoms: *n* = 78).

In the OCD group only, the association between global scores for fear, incompleteness, and disgust, and hoarding symptoms, comorbid tic disorder, autism spectrum disorder, and autistic traits, were assessed via independent samples t-tests (and Mann–Whitney *U* tests) and zero-order Pearson and Spearman correlations. The statistical analyses were carried out in SPSS v. 23.0 and R Studio version 1.1.447.

## Results

### Group comparisons

**Fig. 1:**
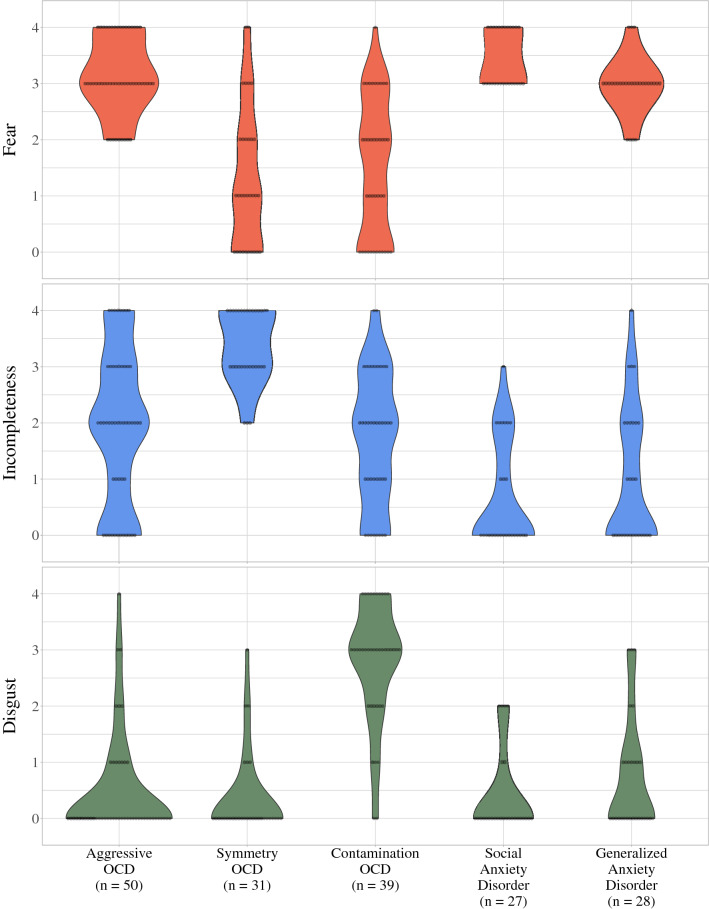
Violin plots and each individual rating across groups for fear, incompleteness, and disgust. *OCD* obsessive–compulsive disorder

**Fig. 2:**
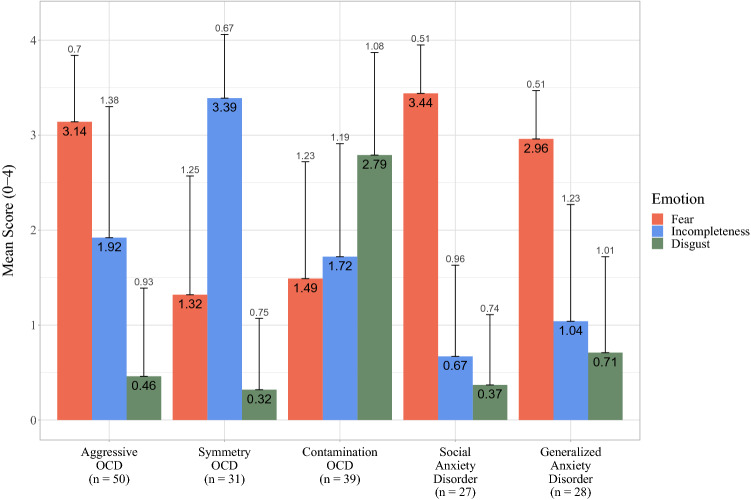
Means and standard deviations for fear, incompleteness, and disgust across groups. *OCD* obsessive–compulsive disorder

Figure 1 presents the distribution of global scores for fear, incompleteness, and disgust across the diagnostic/symptom groups. Means and standard deviations for these scores are presented in Fig. 2.

Diagnostic/symptom group had a statistically significant effect on the degree to which fear was rated as accompanying symptoms (*F*[4, 168] = 34.66, *p* < 0.001, *η*_p_^2^ = 0.45). Follow-up tests showed that participants with aggressive OCD (*M* = 3.16, SE = 0.13) had higher fear scores than those with symmetry OCD (*M* = 1.40, SE = 0.17, *p* < 0.001), and contamination OCD (*M* = 1.49, SE = 0.15, *p* < 0.001). Participants with SAD (*M* = 3.35, SE = 0.19, *p* < 0.001) and GAD (*M* = 2.93, SE = 0.18, *p* < 0.001) had higher fear scores than those with symmetry and contamination OCD. No other significant group differences emerged.

Diagnostic/symptom group also showed a significant effect on the degree to which incompleteness was rated as accompanying symptoms (*F*[4, 168] = 20.55, *p* < 0.001, *η*_p_^2^ = 0.33). Participants with symmetry OCD (*M* = 3.37, SE = 0.21) had higher incompleteness scores than all other groups: aggressive OCD (*M* = 1.92, SE = 0.17, *p* < 0.001); contamination OCD (*M* = 1.74, SE = 0.19, *p* < 0.001); SAD (*M* = 0.67, SE = 0.24, *p* < 0.001); and GAD (*M* = 1.03, SE = 0.23, *p* < 0.001). Participants with aggressive OCD had higher scores than those with SAD (*p* < 0.001) and GAD (*p* = 0.002). Participants with contamination OCD had higher scores than those with SAD (*p* = 0.001) and GAD (*p* = 0.018). No other significant group differences emerged.

Last, diagnostic/symptom group had a significant effect on the degree of disgust during symptoms (*F*[4, 168] = 42.44, *p* < 0.001, *η*_p_^2^ = 0.55). Participants with contamination OCD (*M* = 2.85, SE = 0.15) had higher scores than all other groups: aggressive OCD (*M* = 0.50, SE = 0.13, *p* < 0.001); symmetry OCD (*M* = 0.38, SE = 0.17, *p* < 0.001); SAD (*M* = 0.25, SE = 0.19, *p* < 0.001); GAD (*M* = 0.63, SE = 0.18, *p* < 0.001). No other group significant differences emerged.

For fear and disgust, non-parametric Kruskal–Wallis *H* tests showed identical results for all comparisons. For incompleteness, identical results emerged except that there was no significant difference between the contamination OCD group and the GAD group; however, the difference approached significance (*p* = 0.052).

### Symptom dimension severity

Table 2 presents the results for the three multiple linear regressions with OCD dimension severity for aggressive, symmetry, and contamination symptoms, respectively, as the dependent variable and dimension-specific ratings for fear, incompleteness, and disgust within each of these dimensions as the independent variables. All overall models were statistically significant: aggressive (*F*[5, 78] = 9.73, *p* < 0.001, *R*^2^ = 0.384); symmetry (*F*[5, 74] = 15.11, *p* < 0.001, *R*^2^ = 0.505); and contamination (*F*[5, 72] = 2.38, *p* = 0.047, *R*^2^ = 0.142). Higher ratings of fear and incompleteness during aggressive symptoms were significantly associated with more severe symptoms within this dimension. Likewise, higher levels of incompleteness during symmetry symptoms and disgust during contamination symptoms were associated with more severe symptoms within each of these respective symptom dimensions. Female gender was significantly associated with higher levels of symmetry symptoms; no other significant associations were found for age and gender. Full results are presented in Table 2.

**Table 2:** Results of the three multiple linear regressions with OCD symptom dimension severity as the dependent variable and fear, incompleteness, and disgust ratings within that dimension as independent variables

	Aggressive symptoms *n* = 84				Symmetry symptoms *n* = 80				Contamination symptoms *n* = 78			
	*B*	95% CI	*β*	*p*	*B*	95% CI	*β*	*p*	*B*	95% CI	*β*	*p*
Fear (dimensional)	1.67	1.07, 2.27	0.52	< 0.001	0.23	− 0.22, 0.68	0.10	0.312	0.15	− 0.34, 0.64	0.07	0.542
INC (dimensional)	0.44	0.09, 0.80	0.23	0.015	1.96	1.44, 2.48	0.64	< 0.001	0.38	− 0.07, 0.84	0.19	0.099
DISG (dimensional)	0.09	− 0.53, 0.72	0.03	0.766	− 0.35	− 1.09, 0.40	− 0.09	0.355	0.67	0.17, 1.16	0.30	0.009
Male versus female	0.33	− 0.71, 1.38	0.06	0.526	− 0.99	− 1.92, − 0.07	− 0.18	0.355	0.67	− 1.04, 1.29	0.02	0.830
Age in years	− 0.10	− 0.29, 0.09	− 0.10	0.301	− 0.06	− 0.22, 0.10	− 0.18	0.355	0.67	− 0.21, 0.22	0.01	0.946

Age and gender were included as covariates

*INC* incompleteness, *DISG* disgust

### Relationships with hoarding, tic disorder, and autism

Within the OCD group, the zero-order Pearson correlations between self-reported hoarding symptoms (OCI-CV; *n* = 100) and global scores for fear (*r* = 0.01) and disgust (*r* = − 0.03) were non-significant, but a significant correlation emerged in relation to incompleteness (*r* = 0.29, *p* < 0.001). Independent samples *t* tests showed no statistically significant differences between OCD participants with (*n* = 35) versus without (*n* = 88) a history of tic disorder for fear (*t*[121] = − 1.08, *p* = 0.281), incompleteness (*t*[121] = 1.13, *p* = 0.260), or disgust (*t*[121] = − 0.46, *p* = 0.644), nor for participants with (*n* = 9) versus without autism (*n* = 115): fear (*t*[122] = − 1.61, *p* = 0.110); incompleteness (*t*[122] = 0.49, *p* = 0.626); disgust (*t*[122] = − 0.39, *p* = 0.699). Similar non-significant results were found using Mann–Whitney *U* tests. There were no statistically significant correlations between parent-reported autistic traits (AQ10; *n* = 69) and fear (*r* = 0.01; Spearman’s rank order correlation = 0.10), incompleteness (*r* = 0.06; Spearman’s rank order correlation = 0.03), or disgust (*r* = − 0.03; Spearman’s rank order correlation = − 0.00).

## Discussion

To better understand the onset and maintenance of the heterogeneous symptoms of OCD, emotion-related processes beyond fear and anxiety need to be investigated [53]. The present study was carried out with this aim and addressed several gaps in the extant literature on emotional motivators in OCD, particularly in relation to children and adolescents. First, we moved beyond trait-level assessment of emotion and investigated the degree to which specific emotions occur during symptoms of OCD. Second, we drew upon two previously separate programs of research, the first focusing on fear (harm avoidance) and incompleteness [16], and the second on disgust [54], to conjointly examine these emotions in youth with OCD in comparison to youth with GAD and SAD. Third, for the OCD group, we examined the relationship between these emotions and the presence of tic disorder, autism, and hoarding symptoms.

As expected, and in line with the dominant cognitive behavioral models of OCD [11], participants with OCD reported elevated levels of fear during symptoms within the aggressive, symmetry, and contamination-related dimensions, with the presence of fear being most strongly elevated during symptoms in the aggressive dimension. This is not surprising given that aggressive symptoms are characterized by obsessions about possible harm befalling oneself or a loved one, often paired with checking and/or idiosyncratic rituals to reduce the likelihood of harm. Also consistent with expectations, youth with SAD and GAD reported that fear was the primary emotion experienced during the respective symptoms of these disorders. These results provide preliminary support for the argument that OCD is more similar than different from the anxiety disorders—at least in this age range. However, in the present study (higher) levels of incompleteness during symptoms differentiated youth with OCD (overall and for the aggressive dimension subtype) from those with SAD and GAD. Also, within the aggressive OCD symptom dimension, incompleteness was both elevated and associated with symptom severity. This is the first study to undertake a comparison between OCD and anxiety disorders assessing experience of different emotions during symptoms, as opposed to the use of trait-like measures of emotional proneness given contemporaneously to OCD symptom measures (interview or self-report). The present results provide further support that, and in contrast to the anxiety disorders, OCD is a more heterogeneous condition at the emotion level, which may help explain why OCD symptoms can be so varied in their topography. Although further studies are needed, the present results suggest that incompleteness, and the mechanisms that might underpin this emotion-like state, warrant further study in this age range, and as part of a large program of research to identify factors that may add (and improve upon) current etiological models of, and treatments for, pediatric OCD.

It is important to note that although incompleteness was elevated across all OCD symptom dimensions, the strongest and most unique association was found with symptoms from the symmetry dimension. Indeed, within this dimension, almost all participants with OCD reported moderate to extreme levels of incompleteness during symptoms, and incompleteness, but not fear or disgust, was associated with the severity of symptoms. Thus, in future studies examining the relationship between incompleteness and OCD, it is important that the OCD symptoms be assessed at both the overall (disorder) and at the symptom dimension level.

The limitations of a fear-centric model of OCD were also evident in relation to OCD symptoms within the contamination dimension, where disgust was the emotion that participants most often reported as occurring during these symptoms. Although disgust by some participants was reported during symptoms from the other OCD symptom dimensions, and to some extent during symptoms of SAD and GAD, it was most strongly related to symptoms within the contamination dimension. Previous studies have found a relatively weak relationship between disgust proneness, measured at the trait-level via self-report questionnaires, and the overall severity of OCD symptoms in clinically referred youth and adult samples [22, 30]. The difference between these and the present findings can be partly attributed to our use of an interview-based assessment of emotion involvement during OCD symptoms and of OCD at the dimensional level. The present findings, while preliminary in nature, suggest that disgust may play a clearer role in the contamination symptoms of OCD when this emotion is measured at the state-level (i.e., as an emotion that accompanies symptoms) than at the trait-level. It is also important to note that although disgust was clearly elevated within the contamination symptom dimension, the overall level of symptom variance within this dimension was weakly explained by the presence of fear, incompleteness, and disgust. These results suggest that other emotion-related motivators may play a role in contamination symptoms, or that some complex interaction between several different emotions is present.

As described above, we attempted to address variation in emotion-involvement across subtypes of OCD defined by its major symptom dimensions. Another potentially important subtype is tic-related OCD, which is found in 10–40% of youth with OCD and is associated with differences in gender distribution, onset, heritability, and a poor response to SSRIs [42, 55]. We found no differences in the severity of fear, incompleteness, and disgust in youth with versus without this subtype of OCD. It is also important to consider influences arising from other comorbid disorders. For example, there is ample evidence in the literature of high levels of comorbidity between OCD and autism [56]. Contrasting with the view that incompleteness may help explain this comorbidity [57], we found no evidence of a relationship between incompleteness and the presence versus absence of a comorbid diagnosis of autism spectrum disorder, or to the severity of the young person’s autistic traits. Finally, while hoarding is now its own disorder in DSM-5, it is still possible for hoarding to occur within the context of OCD symptoms [43]. Incompleteness and not just right experiences have been found to be associated with hoarding symptoms in adults with OCD [58] and a small but significant correlation was found also in our study, which adds to the literature about potential mechanisms involved in hoarding symptoms and how these symptoms may be linked to OCD.

Exposure-based CBT is the recommended first-line treatment for pediatric OCD and the rationale for this treatment draws heavily on a fear-centric model in which symptom change is assumed to occur as a result of therapist and patient-guided exposures to fear-eliciting stimuli/situations during which the urge to engage in compulsive behaviors is resisted. It is generally assumed that such exposures (in session and as homework) bring about a gradual extinction or habituation of the fear response and an overall attenuation of OCD symptoms. Recent models have challenged the role of habituation in symptom improvement but still highlight the role of fear expectancies [59]. Given the present results, with incompleteness and to some extent disgust emerging as equally important as fear in relation to pediatric OCD, it is reasonable to ask whether the efficacy of CBT for OCD is partly dependent upon the emotions that underpin symptoms. A study carried out on adults with blood/injury phobias found that disgust appeared to decline more slowly than fear with repeated exposures to distress-provoking stimuli, and that additional exposure sessions may be required [60]. Recently, using a subset of the OCD participants in this study for whom pre- and post-treatment data were available, we found that high levels of incompleteness or disgust during OCD symptoms at pre-treatment predicted a poorer response to treatment involving exposure plus response prevention with or without concomitant pharmacotherapy [24]. We are unaware of any experimental studies examining the effects of exposure on incompleteness-driven OCD. It has been suggested that habit reversal techniques may be needed to improve outcomes [20], but to the best of our knowledge, this is still an empirically untested hypothesis. Whether responsiveness to SSRIs and other pharmacological agents is dependent upon emotion involvement in symptoms is also an area of research that could help better elucidate treatment response in pediatric OCD. More research is needed, not only across different symptom dimensions of pediatric OCD, but also in relation to the emotional processes that underpin these symptoms.

Although the study benefits from the inclusion of a large and diverse sample of youth with OCD, clinical comparison groups, and the use of structured interviews to assess emotion during symptoms, the findings need to be viewed within the context of certain limitations. First, emotions are complex phenomena involving more than a person’s verbal report, and the current findings are based solely on structured interviews wherein patients were asked to rate the degree to which these specific emotions were experienced during symptom episodes. It is possible that the emotions described during the OC-CDI interview induced participants to conceptualize the emotional content accompanying their symptoms according to these descriptions. While an individual’s subjective experience of emotion is a valid and fundamental component in clinical research [61], studies employing psychophysiological, brain imaging, symptom-provocation, and experience sampling methods should be used to build on the results reported here. Novel measures may be used alongside the expanded OC-CDI interview used here, as it shows potential as a valid measure of emotion involvement in OCD. Indeed, the present results further support its construct validity, which previously has been demonstrated in relation to self-reported trait-levels of incompleteness and harm avoidance [50]. Second, the study was carried out with clinically referred children and adolescents and these findings may not extend to non-clinical samples or adults. Furthermore, substantial proportions of youth with OCD in this study also fulfilled diagnostic criteria for SAD and GAD, which obscures inferences regarding group differences. Third, emotional-involvement in symptoms was measured retrospectively, which can introduce certain biases and limit the inferences that can be drawn [62]. Fourth, fear, disgust, and incompleteness are likely only a subset of the emotions involved in OCD. Fifth, our use of the symptom dimension model to assess OCD symptoms may have been overly restrictive. It is possible that emotion-involvement is a more solid ground on which to build a multidimensional model of OCD (see [55]), but this hypothesis could not be thoroughly tested given the design of the present study.

In summary, we found that the emotions that accompany the symptoms of pediatric OCD extended beyond fear, to include incompleteness and disgust, with the level of involvement of these emotions varying as a function of the symptom dimension that characterized the child’s OCD presentation. Importantly, our results showed that incompleteness was as central to OCD as fear, and more specific as it differentiated youth with OCD from those with SAD and GAD. Disgust was also specific to OCD but primarily in relation to symptoms in the contamination dimension. While further studies are needed, the present results suggest that researchers and clinicians alike should consider models of OCD that more clearly account for emotional motivators other than fear.
